# Dependence of NPPS creates a targetable vulnerability in RAS-mutant cancers

**DOI:** 10.1038/s41401-024-01409-2

**Published:** 2024-11-06

**Authors:** Rui-xue Xia, Pei-chen Zou, Jun-ting Xie, Ya-bin Tang, Miao-miao Gong, Fu Fan, Ayinazhaer Aihemaiti, Yu-qing Liu, Ying Shen, Bin-bing S. Zhou, Liang Zhu, Hui-min Lei

**Affiliations:** 1 Key Laboratory of Pediatric Hematology and Oncology Ministry of Health, Pediatric Translational Medicine Institute, Shanghai Children’s Medical Center, Shanghai Jiao Tong University School of Medicine, Shanghai, 200127 China; 2 Department of Pharmacology and Chemical Biology, College of Basic Medical Sciences, Shanghai Jiao Tong University School of Medicine, Shanghai, 200025 China

**Keywords:** RAS-mutant cancers, NPPS, HK1, glycolysis

## Abstract

RAS is the most frequently mutated oncoprotein for cancer driving. Understanding of RAS biology and discovery of druggable lynchpins in RAS pathway is a prerequisite for targeted therapy of RAS-mutant cancers. The recent identification of KRAS^G12C^ inhibitor breaks the “undruggable” curse on RAS and has changed the therapy paradigm of KRAS-mutant cancers. However, KRAS mutations, let alone KRAS^G12C^ mutation, account for only part of RAS-mutated cancers. Targeted therapies for cancers harboring other RAS mutations remain the urgent need. In this study we explored the pivotal regulatory molecules that allow for broad inhibition of RAS mutants. By comparing the expression levels of nucleotide pyrophosphatase (NPPS) in a panel of cell lines and the functional consequence of increased NPPS expression in RAS-mutant cells, we demonstrated that cancer cells with various kinds of RAS mutations depended on NPPS for growth and survival, and that this dependence conferred a vulnerability of RAS-mutant cancer to treatment of NPPS inhibition. RAS-mutant cells, compared with RAS-wildtype cells, bored and required an upregulation of NPPS. Transcriptomics and metabolomics analyses revealed a NPPS-dependent hyperglycolysis in RAS-mutant cells. We demonstrated that NPPS promoted glucose-derived glycolytic intermediates in RAS-mutant cells by enhancing its interaction with hexokinase 1 (HK1), the enzyme catalyzing the first committed step of glycolysis. Pharmacological inhibition of NPPS-HK1 axis using NPPS inhibitor Enpp-1-IN-1 or HK1 inhibitor 2-deoxyglucose (2-DG), or genetic interfere with NPPS suppressed RAS-mutant cancers in vitro and in vivo. In conclusion, this study reveals an unrecognized mechanism and druggable lynchpin for modulation of pan-mutant-RAS pathway, proposing a new potential therapeutic approach for treating RAS-mutant cancers.

## Introduction

The RAS family of proto-oncogenes, including KRAS, NRAS and HRAS, are prevalently mutated in human cancers, which leads to the initiation and progression of tumors [1–4]. Among these, KRAS variants are the most common RAS mutations, with codon 12 being the predominant mutated spot [2, 4]. Despite decades of effort, the quest to directly inhibit RAS has been mostly unsuccessful until recent years. Currently, a number of KRAS^G12C^-specific covalent inhibitors, including sotorasib and adagrasib, have received regulatory approval [5–9]. Nevertheless, these inhibitors encounter severe challenges, such as variable responses and the rapid emergence of drug resistance [10, 11], highlighting the need to develop more effective treatments. Furthermore, KRAS^G12C^ inhibitors are not applicable to tumors driven by other oncogenic RAS variants. Therefore, efforts to discover therapeutics that allow for broad inhibition of RAS mutants are ongoing. Hence, pinpointing the pivotal regulatory molecules of pan-RAS mutations is a significant challenge.

The reprogramming of cellular metabolism to meet the energy and biomass requirements for uncontrolled proliferation is a characteristic hallmark of cancer [12–14]. Our previous studies have revealed that the metabolism of cancer and essential metabolic enzymes present a novel aspect of the mechanism for drug resistance [15–17]. Oncogenic RAS deregulates critical metabolic pathways, particularly glycolysis, glutaminolysis, autophagy and macropinocytosis [18]. Consequently, the interplay between the oncogenic RAS and metabolic reprogramming provides an alternative strategy for pharmacological targeting of RAS-driven cancers.

In this study, we demonstrated that cancer cells with various kinds of RAS mutations were regulated by and dependent on nucleotide pyrophosphatase (NPPS) for growth and survival, and that this dependence conferred a vulnerability of RAS-mutant cancer to pharmacological and genetic treatment of NPPS inhibition. Mechanistically, in RAS-mutant cells, NPPS interacted with the N-terminal region (aa 22–474) of hexokinase 1 (HK1) and promoted glycolysis and cancer growth and survival, independent of its canonical nucleotide-metabolizing activity. Our research reveals an unrecognized mechanism and druggable lynchpin for the modulation of the pan-mutant-RAS pathway, proposing a new potential therapeutic approach for treating RAS-mutant cancers; moreover, an alternative metabolism-regulating function of NPPS in glycolysis other than its canonical nucleotide-catalytic activity is demonstrated.

## Materials and methods

### Cell lines

The human cancer cell lines HCC827 (KRAS^WT^), NCI-H292 (KRAS^WT^), A549 (KRAS^G12S^), NCI-H1299 (NRAS^Q61K^), NCI-H460 (KRAS^Q61H^), PANC-1 (KRAS^G12D^), HCT116 (KRAS^G13D^), SW480 (KRAS^G12V^) and the human embryonic kidney cell line HEK293T were obtained from American Type Culture Collection (ATCC). The human cancer cell line PA-TU-8902 (KRAS^G12V^) was obtained from Deutsche Sammlung von Mikroorganismen und Zellkulturen (DSMZ). The human lung epithelial cell line BEAS-2B and the human pancreatic duct epithelial-like cell line hTERT-HPNE were obtained from FuHeng Biology. The human cancer cell line NCI-H2030 (KRAS^G12C^) was obtained from Zhong Qiao Xin Zhou Biotechnology. The human cancer cell line PC9 (KRAS^WT^) was a kind gift from Dr. Q. Dong (China State Key Laboratory of Oncogenes and Related Genes). All the cell lines were authenticated routinely via short tandem repeat (STR) DNA profiling and tested for mycoplasma contamination every 6 months using MycoBlue Mycoplasma Detector (Vazyme).

BEAS-2B, HEK293T, HPNE, PANC-1 and PA-TU-8902 cells were propagated in DMEM medium (BasalMedia), A549 cells were propagated in F-12K medium (BasalMedia), HCT116 cells were propagated in McCoy’s 5a medium (BasalMedia), SW480 cells were propagated in Leibovitz’s L-15 medium (BasalMedia), and HCC827, PC9, NCI-H292, NCI-H1299, NCI-H460 and NCI-H2030 cells were propagated in RPMI-1640 medium (Gibco). All media were supplemented with 10% fetal bovine serum (Biological Industries), 1% penicillin‒streptomycin (BasalMedia) and 1% GlutaMax (BasalMedia).

### Construction of DOX-inducible shNPPS cell lines

A lentivirus system was used to construct doxycycline-induced shNPPS cell lines. The inducible shRNA plasmids targeting NPPS (GV307 vector) were obtained from GeneChem, and their sequences were based on siNPPS #4 and #5. In brief, lentiviruses were generated in HEK293T cells by cotransfecting the shNPPS plasmid and virus-packaging vectors (pSPAX2 and pMD2.G) and then the viral supernatants were harvested after 24 h and 48 h to infect the target cells. Positive cells were selected with puromycin (1 μg/mL) after 48 h of infection.

### Isogenic H292 cells

Using homologous recombination (HR)-mediated gene knock-in based on CRISPR/Cas9 technology, we inserted the KRAS p.G12C gene variant into the H292 cell line. In brief, the KRAS knock-out plasmid (pX459-sgKRAS) and the KRAS G12C donor plasmid (pUC19-KRAS^G12C^) were kindly provided by Dr. Y. Shen (Shanghai Jiao Tong University School of Medicine). H292 cells were seeded in 6-well plates. The next day, the cells were cotransfected with the sgKRAS plasmid and the donor plasmid using Lipofectamine 3000 (Invitrogen). After 48 h, cell media were supplemented with puromycin (1 μg/mL), and single-cell colonies were subsequently established via dilution in 96-well plates. After 10 days, single cell clones were transferred to 24-well plates and expanded. For sequence analysis, genomic DNA was extracted from single cell clones using the Genomic DNA Mini-Preps Kit (Sangon Biotech) and PCR products were amplified using the forward primer 5ʹ-AGCGTCGATGGAGGAGTTTG-3ʹ and the reverse primer 5ʹ-GACCCTGACATACTCCCAAGG-3ʹ. The PCR products were subsequently sequenced using the forward primer (BioSune Biotechnology) and sequence analysis was conducted using SnapGene software.

### siRNA and plasmid transfection

For siRNA transfection, cells were plated at a confluence of 30%–40% when they were transfected with a specific-targeted siRNA duplex using Lipofectamine 3000 (Invitrogen) according to the manufacturer’s instructions. As control, cells were transfected with a noncoding siRNA (NC). The final concentration of the siRNAs was 20 nM. The siRNAs were obtained from Shanghai GenePharma. The NPPS siRNA sequences from 5ʹ to 3ʹ were as follows:

siNPPS #4: -AGATAAATACTATTCATTT-

siNPPS #5: -AGCTTCTATCAACAAAGAA-

For plasmid transfection, cells were plated in 6 cm dishes at a confluence of 60%–80% when they were transfected with 500 ng of plasmid using Lipofectamine 3000 (Invitrogen) according to the manufacturer’s instructions. The cells were transfected with the empty vector as a negative control. Plasmids for human NPPS (NM_006208.3), human KRAS (NM_004985), KRAS G12C (G12C mutation), KRAS G12D (G12D mutation), and KRAS Q61H (Q61H mutation) with a C-terminal 3 × Flag (CV702 vector), and plasmids for human HK1 (NM_000188.3), HK1-∆MBD (1–21 aa deletion), HK1-∆N’ (22–474 aa deletion), and HK1-∆C (475–917 aa deletion) with a C-terminal HA (GV366 vector) were constructed by Shanghai GeneChem.

### Cell growth and cell viability assays

A total of 3000–4000 cells were seeded in 96-well plates. The next day, the cells were transfected with siRNAs or treated with drugs at the indicated dosages. Cell growth was monitored by IncuCyte ZOOM live cell analysis system (Essen BioScience) every 4 h for 72‒96 h. Cell viability was determined via Cell Counting Kit-8 (CCK-8; Proteintech Group) according to the manufacturer’s instructions.

For some experiments, the cells were fixed with 4% paraformaldehyde and stained with 0.1% crystal violet after the indicated treatment. The plates were scanned by Epson Scanner (Seiko Epson Corporation). The crystal violet in the plates was dissolved in 10% acetic acid, and the optical density at a wavelength of 600 nm (*OD*_600_) was detected on a microplate reader (Thermo Fisher Scientific).

### Inhibitors and antibodies

The following compounds were used in this study: 2-Deoxy-*D*-glucose (Selleck, S4701), Enpp-1-IN-1 (Selleck, S0501), Sotorasib (Aladdin, S414206), and Doxycycline Hyclate (Selleck, S4163).

The following antibodies were used in this study: NPPS (Abcam, ab40003), NPPS (Abcam, ab223268), HK1 (Cell Signaling Technology, #2024), pERK1/2 (Cell Signaling Technology, #4370), ERK1/2 (Cell Signaling Technology, #4695), pAKT (S473) (Cell Signaling Technology, #4060), AKT (Cell Signaling Technology, #4691), HA (Cell Signaling Technology, #3724), β-actin (Proteintech Group, 66009-1-Ig), and Flag (Sigma‒Aldrich, F1804).

### Western blot analysis

The cells or tumor samples were lysed on ice with RIPA lysis buffer (Beyotime) supplemented with protease inhibitor (Beyotime). The protein concentration was measured by BCA Protein Assay Kit (Thermo Fisher Scientific) and adjusted to approximately 2 μg/μL. Samples containing 40 μg of protein were separated by SDS‒PAGE and then transferred to PVDF membranes (Millipore) followed by blockage with 5% nonfat milk for 1.5 h at room temperature. The membranes were then incubated with primary antibodies at 4 °C overnight. The next day, the membranes were incubated with HRP-conjugated secondary antibodies (Beyotime) for 1.5 h at room temperature. Immunoblots were developed via enhanced chemiluminescence (ECL) (Thermo Fisher Scientific) and scanned with the Odyssey Fc imaging system (LI-COR Biosciences).

### Coimmunoprecipitation (Co-IP)

The cells were lysed on ice with NP-40 lysis buffer (Beyotime) supplemented with protease inhibitor (Beyotime). The protein concentration was measured by BCA Protein Assay Kit (Thermo Fisher Scientific) and samples containing 1–1.5 mg of protein were used for immunoprecipitation.

For IP of endogenous proteins, lysates were incubated with anti-NPPS or anti-HK1 at 4 °C with rotation overnight. The next day, Protein A/G Magnetic Beads (Millipore) were added to the lysates to capture the immunocomplex for 3 h.

For IP of exogenous Flag-NPPS, lysates were incubated with anti-FLAG M2 Magnetic Beads (Sigma–Aldrich) at 4 °C with rotation overnight.

After incubation, the beads were washed for 5 times and resuspended in 2 × SDS loading buffer (Beyotime) followed by boiling at 100 °C for 5 min. Co-IP proteins were evaluated via Western blot analysis.

### Protein mass spectrometry

The immunoprecipitates pulled down with anti-NPPS from H292 samples were used for protein mass spectrometry. All MS experiments were performed on a QE-Plus mass spectrometer connected to an Easy-nLC2000 via an Easy Spray (Thermo Fisher Scientific). The ion spectra were analyzed using PEAKS 8.0 (Bioinformatics Solutions) for processing, de novo sequencing and database searching. The resulting sequences were searched through the UniProt Human Proteome database (downloaded on May 5th, 2018). For all the experiments, these settings yielded an FDR of < 1% at the peptide-spectrum match level.

### RNA-seq analysis

For RNA-seq, RNA was isolated using TaKaRa MiniBEST Universal RNA Extraction Kit (TaKaRa) and then reverse transcribed into cDNA by PrimeScript™ RT reagent Kit (TaKaRa). The samples were library prepped and sequenced via the Illumina HiSeq 4000 platform by BGI Genomics. Differentially expressed gene (DEG) analysis and gene set enrichment analysis (GSEA) were performed using an online-based software Dr. TOM (BGI).

### LC‒MS/MS analysis

For untargeted metabolomics, cells were cultured in 6-well plates and collected in 1.5 mL centrifuge tubes. Metabolites were then extracted with 80% ice-cold methanol followed by vortexing for 5 min. After centrifugation at 20,000 × *g* and 4 °C for 15 min, the supernatant was transferred and evaporated to dryness using a vacuum centrifuge. The samples were resuspended in 200 µL of 50% (*v*/*v*) acetonitrile prior to LC‒MS/MS analysis.

LC‒MS/MS was performed using the ExionLC AD UPLC system (SCIEX) coupled with a TripleTOF 6600 Plus mass spectrometer (SCIEX). Data were acquired using Analyst 1.8.4 software (SCIEX). Metabolite identification and quantitation were performed using the SCIEX OS 1.7 software (SCIEX).

### SIRM analysis

For glucose metabolism SIRM analysis, the cells were cultured in 6-well plates and the cell medium was changed to glucose-free RPMI-1640 medium supplemented with 10% FBS and 11 mM U-^13^C_6_-glucose (Cambridge Isotope Laboratories) 6 h before sample collection. The sample pretreatment process was referred to the LC‒MS/MS analysis. The samples were analyzed via a TripleTOF 6600 plus mass spectrometer (SCIEX) and the data were acquired using SCIEX OS 1.7 software (SCIEX). The incorporation of ^13^C from U-^13^C_6_-glucose induced an intensity shift from the unlabeled position (m + 0) to the labeled position (m + n, where ‘n’ represents the number of incorporated ^13^C atoms). The natural isotope abundance was corrected by the AccuCor R package (https://github.com/XiaoyangSu/IsotopeNatural-Abundance-Correction).

### Seahorse assay

The cells were attached to culture plates at a density of 20,000 cells per well. The extracellular acidification rate (ECAR) was measured with a Seahorse XFe96 Analyzer (Agilent Technologies) according to the manufacturer’s instructions, in the presence of the following compounds: 10 mM glucose, 1 μM oligomycin and 50 mM 2-DG.

### HK activity assay

HK activity assays were performed using the Hexokinase Assay Kit (Enzyme-linked Biotechnology) according to the manufacturer’s instructions.

### Combination effect analysis

The effects were evaluated using a combination index (CI) calculation in accordance with the Chou-Talalay method. The data were analyzed using the CompuSyn software (CompuSyn Inc.): CI = 1, additive effect. CI > 1, antagonistic effect. CI < 1, synergistic effect.

### Xenograft assay

All the animal experiments were approved by the Institutional Animal Care and Use Committee (IACUC) of Shanghai Jiao Tong University School of Medicine and were performed according to the guidelines. For the xenograft assay, approximately 3 × 10^6^ tumor cells were implanted subcutaneously into the flanks of BALB/c *nu*/*nu* athymic mice (female, 5 weeks old). Water containing 2 mg/mL doxycycline (DOX) (in 25 mg/mL sucrose, Sangon Biotech) or the control (25 mg/mL sucrose, Sangon Biotech) was administered to each cohort at one day before tumor inoculation. Tumor volumes were measured with a caliper every 2 or 3 days and calculated as 0.5 × length × width^2^.

### GEO database analysis

**Fig. 1:**
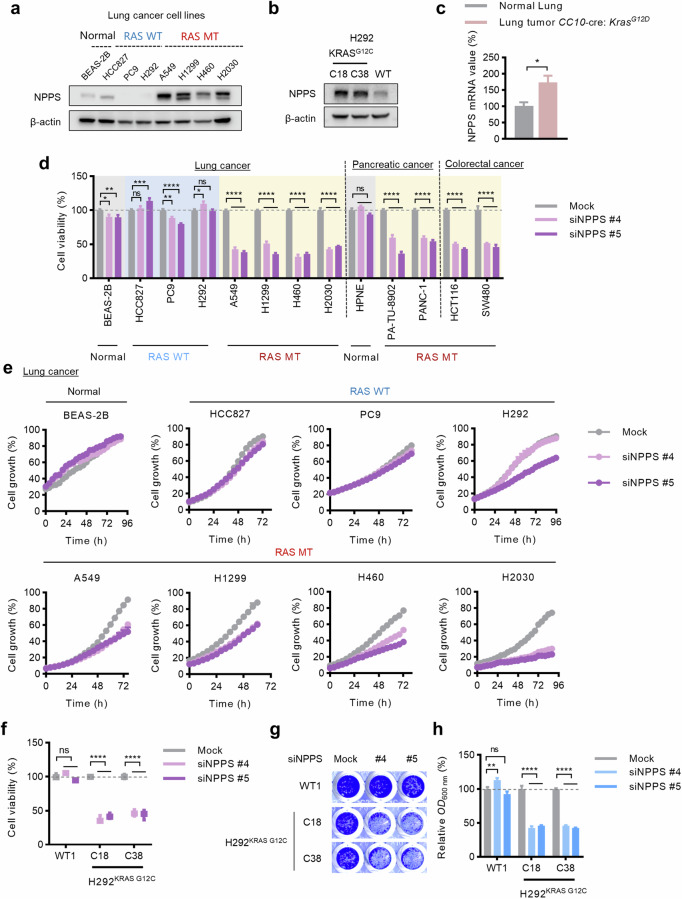
NPPS is selectively crucial for RAS-mutant cells versus RAS-wildtype cells. **a** Protein levels of NPPS in the normal cell line BEAS-2B and cancer cell lines examined by Western blot analysis. **b** Protein levels of NPPS in H292^KRAS G12C^ cells and H292^KRAS WT^ cells examined by Western blot analysis. **c** mRNA levels of NPPS in tumors from *CC10-Cre/LSL-KrasG12D* mice and control murine lung tissues as analyzed via the GEO database analysis (GSE26850). **d** Cell viability of a panel of cell lines after knockdown of NPPS. The cells were transfected with NPPS siRNAs (20 nM) or mock control for 72 h and then cell viability was determined by CCK-8. **e** Growth curves of cell lines after knockdown of NPPS. The cells were transfected with NPPS siRNAs (20 nM) or mock control for 72–96 h and monitored by IncuCyte ZOOM system every 4 h. **f** Cell viability of H292^KRAS WT^ cells and H292^KRAS G12C^ cells after knockdown of NPPS. The cells were transfected with NPPS siRNAs (20 nM) or mock control for 72 h and then cell viability was determined by CCK-8. **g**, **h** Cell growth analysis of H292^KRAS WT^ cells and H292^KRAS G12C^ cells after knockdown of NPPS. The cells were transfected with NPPS siRNAs (20 nM) or mock control for 72 h and then cell growth was measured by the crystal violet assay. Data are shown as the means ± SEMs (*n* = 3 replicates). ns, not significant; *P  <  0.05, **P  <  0.01, ***P  <  0.001, ****P  <  0.0001.

The gene expression profiles shown in Fig. 1c were obtained from the Gene Expression Omnibus (GEO) database (www.ncbi.nlm.nih.gov/geo; GSE26850).

### Statistical analysis

All the data are presented as the means ± SEMs and repeated at least 3 times. Statistical analyses were performed using GraphPad Prism software (version 9.0). Significant differences were conducted by 2-tailed unpaired Student’s *t* test or 1-way or 2-way ANOVA analyses. The statistical parameters can be found in the figures and their corresponding legends. Statistical significance was set as *P* < 0.05.

## Results

### NPPS is selectively crucial for RAS-mutant cells versus RAS-wildtype cells

Although high expression of the NPPS is known to be associated with a poor prognosis in multiple cancers [19], the functions of the NPPS are largely unknown. To further investigate the role of NPPS in cancer, we first examined the endogenous expression levels of NPPS in a panel of cell lines. Compared with human lung epithelial cell line BEAS-2B and RAS-wildtype (WT) cell lines, the protein level of NPPS was greater in the RAS-mutant cancer cell lines (Fig. 1a). To eliminate the impact of genetic background variation among different cell lines, we constructed H292 cells with an endogenous KRAS^G12C^ mutation via homologous recombination (HR)-mediated gene knock-in based on CRISPR/Cas9 technology and the mutation was confirmed via Sanger sequencing (Fig. S1a). Gene set enrichment analysis (GSEA) of RNA-seq datasets from H292^KRAS G12C^ clones C18 and C38 in comparison with the H292^KRAS WT^ clone WT1 demonstrated the upregulation of KRAS signaling in H292^KRAS G12C^ cells (Fig. S1b). Consistent with the results in the cell lines, increased protein levels of NPPS were also observed in H292 isogenic cells with the KRAS^G12C^ mutation compared with H292^KRAS WT^ cells (Fig. 1b). We next introduced three common KRAS mutant variants into HEK293T cells. Compared with the vector control (EV) and KRAS-WT, the expression of oncogenic KRAS variants resulted in the elevated protein levels of NPPS (Fig. S1c), suggesting that NPPS was upregulated in RAS-mutant cells. Furthermore, NPPS was expressed at a higher level in tumors from *CC10-Cre/LSL-KrasG12D* mice than in control murine lung tissues, as analyzed via the Gene Expression Omnibus (GEO) database (GSE26850 [20]) (Fig. 1c). These data indicate that NPPS is upregulated in RAS-mutant cancers.

To determine the functional consequences of increased NPPS expression in RAS-mutant cells, we designed small interfering RNAs (siRNAs) specifically targeting NPPS and confirmed their knockdown efficacy (Fig. S2a). NPPS knockdown with siRNAs in lung cancer, pancreatic cancer and colorectal cancer cell lines selectively suppressed the viability and growth of RAS-mutant cells (Fig. 1d, e, S2b–d). In contrast, knockdown of NPPS had less influence on normal cells and RAS-WT cells. We further generated doxycycline (DOX)-inducible shNPPS cell lines to conditionally interfere with NPPS (Fig. S3a). Knockdown of NPPS using shRNAs also selectively suppressed RAS-mutant cells (Fig. S3b, c). These findings were also observed in H292 isogenic cells (Fig. 1f–h, S3d, e). Collectively, these data suggest that NPPS is preferentially required for RAS-mutant cancer proliferation and survival.

### RAS-mutant cells demonstrate hyperglycolysis in an NPPS-dependent manner

**Fig. 2:**
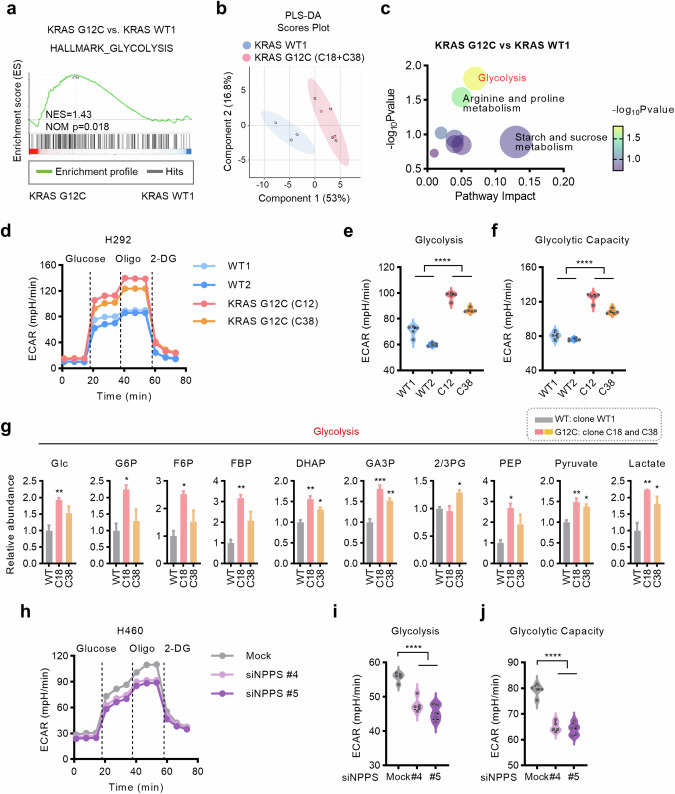
RAS-mutant cells demonstrate hyperglycolysis in an NPPS-dependent manner. **a** GSEA of RNA-seq datasets indicated a glycolysis hallmark in H292^KRAS G12C^ cells compared with H292^KRAS WT^ cells. **b** The PLS-DA model revealed a notable disparity in the metabolic profiles between H292^KRAS WT^ cells and H292^KRAS G12C^ cells. **c** Pathway enrichment analysis based on metabolomics. **d-f** Glycolysis of H292^KRAS WT^ cells and H292^KRAS G12C^ cells measured by analyzing the ECAR using Seahorse XFe96 Analyzer. **g** Relative levels of glycolytic intermediates in H292^KRAS G12C^ cells compared with H292^KRAS WT^ cells determined by LC‒MS/MS analysis. **h–j** Glycolysis of H460 cells after knockdown of NPPS. The cells were transfected with NPPS siRNAs (20 nM) or mock control for 48 h and then the ECAR was measured using the Seahorse XFe96 Analyzer. Data are shown as means ± SEMs (*n* = 3 replicates). ns, not significant; *P  <  0.05, **P  <  0.01, ***P  <  0.001, ****P  <  0.0001.

NPPS is a member of the ecto-nucleotide pyrophosphatase/phosphodiesterase family [19]. However, limited research has been conducted on the additional functions of NPPS in cancers, particularly in cells with RAS mutations. We have demonstrated that the NPPS is crucial for RAS-mutant cancers. To investigate how the NPPS supports RAS-mutant tumor growth, transcriptomics and metabolomics were performed using H292 isogenic cells. GSEA of the RNA-seq datasets from H292^KRAS G12C^ clones C18 and C38 in comparison to H292^KRAS WT^ clone WT1 demonstrated the upregulation of glycolysis signaling in H292^KRAS G12C^ cells (Fig. 2a). Partial least squares discriminant analysis (PLS-DA) and pathway enrichment analysis based on metabolomics revealed a notable disparity in the metabolic profiles of H292 isogenic cells, with significant upregulation of glycolysis in H292^KRAS G12C^ cells (Fig. 2b, c). The live-cell extracellular acidification rate (ECAR) analysis also validated the enhanced glycolysis in RAS-mutant cells (H292KRAS G12C clones C12 and C38) (Fig. 2d–f). These data suggest that glycolysis is upregulated in RAS-mutant cells.

Furthermore, we analyzed the levels of metabolites associated with glycolysis and nucleotide metabolism from metabolomics data. The levels of glycolytic intermediates increased H292^KRAS G12C^ cells versus H292^KRAS WT^ cells, while the metabolites involved in nucleotide metabolism did not show metabolic rewiring (Fig. 2g, S4a). These findings indicate that the function of NPPS in RAS-mutant cells is independent of its canonical nucleotide-metabolizing activity.

We next examined whether NPPS is responsible for glycolysis upregulation in RAS-mutant cells. The elevation of glycolysis in RAS-mutant cells is abrogated by NPPS knockdown, including glycolytic capacity, as determined by ECAR analysis (Fig. 2h–j, S4b–f). Altogether, these results demonstrate that RAS-mutant cells exhibit hyperglycolysis in a manner dependent on NPPS.

### NPPS promotes glycolysis through hyper-interaction with HK1 in RAS-mutant cells

**Fig. 3:**
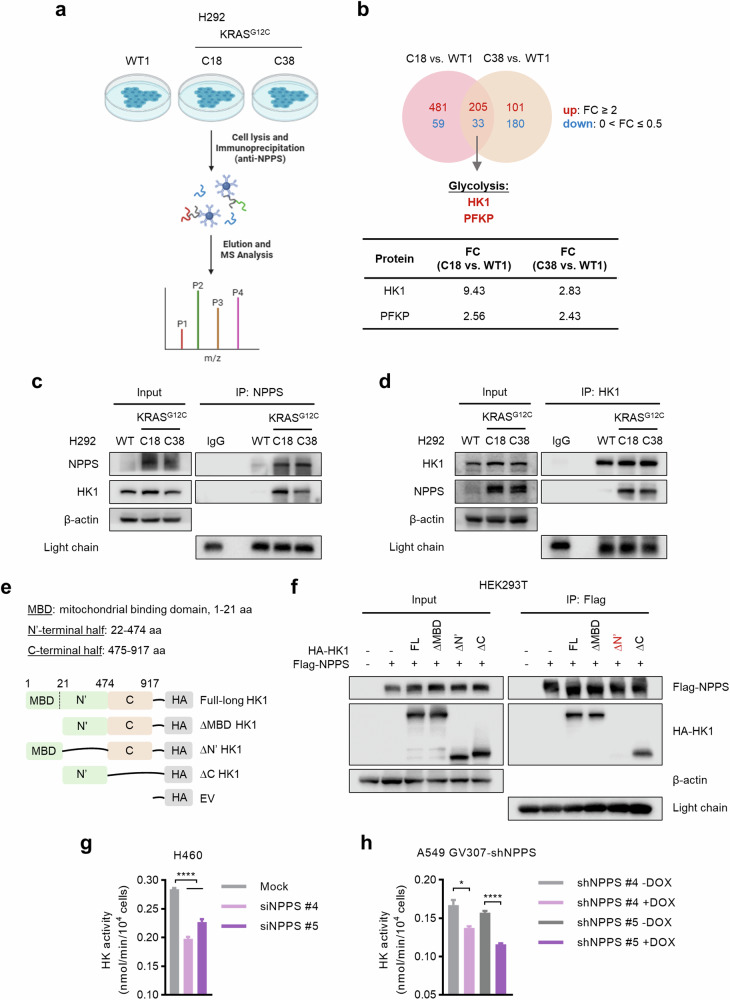
NPPS promotes glycolysis through hyper-interaction with HK1 in RAS-mutant cells. **a** Schematic diagram of the IP‒MS process. **b** Analyses of NPPS-binding proteins based on IP-MS data as indicated in the graph. Endogenous Co-IP assays using anti-NPPS (**c**) and anti-HK1 (**d**) antibodies respectively in H292 isogenic cells. **e** Schematic representation of various HK1 truncations and deletions. **f** Anti-Flag Co-IP assays of the NPPS-HK1 interaction in HEK293T cells cotransfected with Flag-NPPS and HA-HK1 or HK1 truncations and deletions. HK activity analysis after knockdown of NPPS. NPPS was knocked down via siRNAs (20 nM) (**g**) or shRNAs (**h**) and HK activity was measured by the Hexokinase Assay Kit. Data are shown as means ± SEMs (*n* = 3 replicates). ns, not significant; *P  <  0.05, ****P  <  0.0001.

To further investigate the mechanism by which NPPS affected glycolysis, we next performed Immunoprecipitation-Mass spectrometry (IP‒MS) to identify potential NPPS-binding proteins associated with glycolysis in H292 isogenic cells (Fig. 3a). We analyzed the proteins that exhibited concurrent changes in H292^KRAS G12C^ cells (clones C18 and C38) compared with H292^KRAS WT^ cells (clone WT1) and further analyzed the proteins related to glycolysis (Fig. 3b). Finally, there were only two proteins remained: hexokinase 1 (HK1) and phosphofructokinase, platelet (PFKP) (Fig. 3b).

Since HK1 demonstrated a greater fold change, we first considered HK1 as a potential NPPS-binding protein involved in the regulation of glycolysis. HK1, a rate-limiting enzyme in glycolysis, catalyzes the first committed step of glycolysis and is essential for the process [21]. To validate this interaction, endogenous coimmunoprecipitation (Co-IP) assays were performed using anti-NPPS and anti-HK1 antibodies respectively in H292 isogenic cells. Indeed, H292^KRAS G12C^ cells exhibited increased NPPS-HK1 interaction compared with H292^KRAS WT^ cells (Fig. 3c, d). Furthermore, anti-Flag Co-IP assays also revealed an interaction between NPPS and HK1 in HEK293T and PC9 cells expressing Flag-NPPS compared with those expressing the empty vector (EV) (Fig. S5a, b). To gain further insight into the detailed region of HK1 binding to NPPS, we generated various HK1 truncation mutants as indicated (Fig. 3e). The HK1 truncation with aa 22–474 deletion, but not other truncations, failed to interact with NPPS, indicating the requirement of aa 22–474 of HK1 for NPPS binding (Fig. 3f). To investigate how the NPPS-HK1 interaction supports glycolysis in RAS-mutant cells, we performed HK activity assay. Inhibition of NPPS impaired HK enzymatic activity, indicating that NPPS regulated the ability of HK1 to phosphorylate glucose in RAS-mutant cells (Fig. 3g, h). These data suggest that NPPS specifically interacts with the N-terminal region of HK1 (aa 22–474) to promote its catalytic activity in RAS-mutant cells.

To further investigate the role of the NPPS-HK1 interaction in RAS mutant cells, stable isotope-resolved metabolic analysis (SIRM) of U-^13^C_6_-glucose was performed (Fig. S5c). There was a preferential accumulation of glucose-derived, labeled intermediates in the glycolytic pathway compared with other major pathways such as nucleotide synthesis and the TCA cycle in H292^KRAS G12C^ cells (Fig. S5d–f). Notably, the level of glucose-6-phosphate (G6P), which is the product of HK1 in glycolysis, was hyperaccumulated in H292^KRAS G12C^ cells than in H292^KRAS WT^ cells (Fig. S5d). Taken together, these data indicate that NPPS enhances glycolysis through hyperinteraction with HK1 in RAS-mutant cells.

### Targeting the NPPS-HK1-glycolysis axis suppresses RAS-mutant cancers

**Fig. 4:**
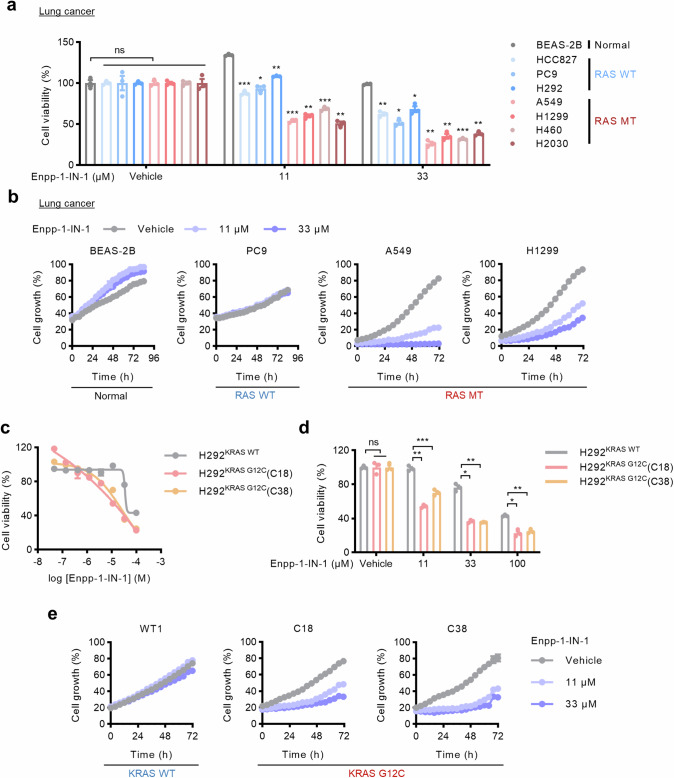
Pharmacological blockade of NPPS selectively suppresses RAS-mutant cells. **a** Cell viability of a panel of cell lines after treatment with Enpp-1-IN-1 at the indicated concentrations. The cells were treated with Enpp-1-IN-1 for 72 h and then cell viability was measured using CCK-8. **b** Growth curves of the cell lines after treatment with Enpp-1-IN-1 at the indicated concentrations. Cells were treated with Enpp-1-IN-1 for 72–96 h and monitored by IncuCyte ZOOM system every 4 h. **c** Effect of Enpp-1-IN-1 on H292^KRAS WT^ cells and H292^KRAS G12C^ cells. The cells were treated with various concentrations of Enpp-1-IN-1 for 72 h and then cell viability was measured using CCK-8. **d** Cell viability of H292^KRAS WT^ cells and H292^KRAS G12C^ cells after treatment with Enpp-1-IN-1 at the indicated concentrations. The cells were treated with Enpp-1-IN-1 for 72 h and then cell viability was measured using CCK-8. **e** Growth curves of H292^KRAS WT^ cells and H292^KRAS G12C^ cells after treatment with Enpp-1-IN-1 at the indicated concentrations. The cells were treated with Enpp-1-IN-1 for 72 h and monitored by IncuCyte ZOOM system every 4 h. Data are shown as means ± SEMs (*n* = 3 replicates). ns, not significant; *P  <  0.05, **P  <  0.01, ***P  <  0.001.

To better elucidate the significance of the NPPS-HK1-glycolysis axis in RAS-mutant cells, a commercially available NPPS inhibitor (Enpp-1-IN-1) was used to inhibit NPPS. Treatment of the cell lines with 11 μM or 33 μM Enpp-1-IN-1 had a minimal effect on normal cells and a modest effect on RAS-WT cells, but showed a significant inhibitory influence on RAS-mutant cells (Fig. 4a, b, S6a–c). Consistently, in H292 isogenic cells, H292^KRAS G12C^ cells exhibited increased sensitivity to Enpp-1-IN-1 compared with H292^KRAS WT^ cells (Fig. 4c). Furthermore, the inhibition of NPPS had a greater effect on the cell viability and growth of H292^KRAS G12C^ cells (Fig. 4d, e). Taken together, these results suggest that pharmacological blockade of NPPS significantly inhibits RAS-mutant cancer cells.

**Fig. 5:**
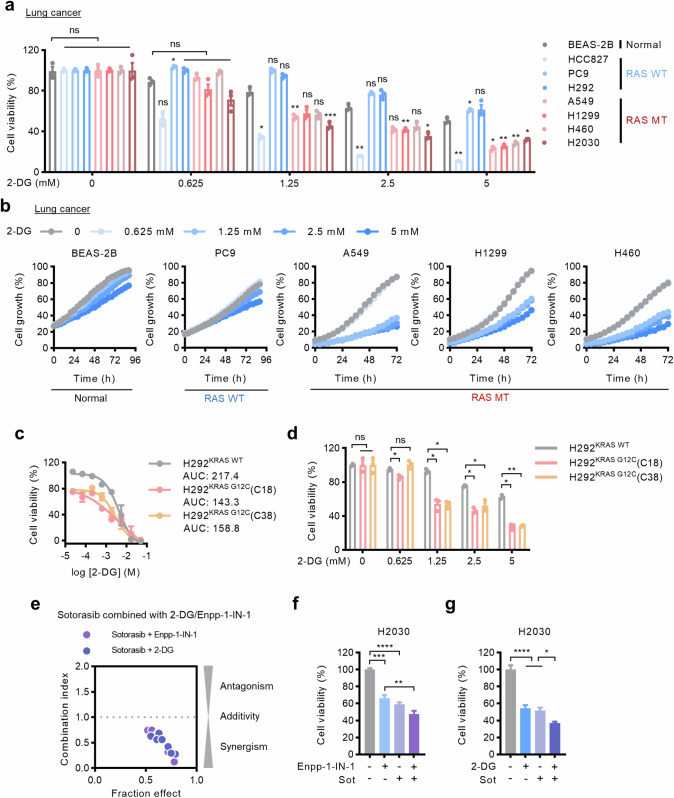
Suppression of HK1 selectively inhibits RAS-mutant cells. **a** Cell viability of the cell lines after treatment with 2-DG at the indicated concentrations. The cells were treated with 2-DG for 72 h and then cell viability was detected using CCK-8. **b** Growth curves of the cell lines after treatment with 2-DG at the indicated concentrations. Cells were treated for 72–96 h and monitored by IncuCyte ZOOM system every 4 h. **c** The impact of 2-DG on H292^KRAS WT^ cells and H292^KRAS G12C^ cells. The cells were treated with various concentrations of 2-DG for 72 h and then cell viability was detected using CCK-8. The area under the curve (AUC) of the drug concentration-effect was used as a quantitative metric for drug activity. **d** Cell viability of H292 isogenic cells after treatment with 2-DG at the indicated concentrations. The cells were treated with 2-DG for 72 h and then cell viability was detected using CCK-8. **e** Synergistic effect of the combination of sotorasib (a KRAS^G12C^ inhibitor) and strategies targeting NPPS-HK1 axis. The CI value (combination index) was calculated as indicated in “Methods” section. Sensitization of Enpp-1-IN-1 (**f**) or 2-DG (**g**) to sotorasib in H2030 cells. In (**f**), Enpp-1-IN-1 was 3.6 μM and Sot was 330 nM. In (**g**), 2-DG was 1.25 mM and Sot was 330 nM. Cell viability assay was performed after cells were treated for 72 h. Data are shown as means ± SEMs (*n* = 3 replicates). ns, not significant; *P  <  0.05, **P  <  0.01, ***P  <  0.001, ****P  <  0.0001.

Next, we further investigated the function of the NPPS-HK1-glycolysis axis in RAS-mutant cells through pharmacological blockade of HK1. 2-Deoxyglucose (2-DG) is both a competitive and a non-competitive inhibitor of HK1 [22]. Treatment of cell lines with 2-DG at concentrations of 1.25, 2.5 and 5 mM resulted in selective inhibition of cell viability and growth in RAS-mutant cells compared to normal cells and most RAS-WT cells, except for HCC827 cells (Fig. 5a, b, S7a–c). Consistently, H292^KRAS G12C^ cells were more sensitive to 2-DG than H292^KRAS WT^ cells and 2-DG exhibited a greater influence on H292^KRAS G12C^ cells (Fig. 5c, d, S7d). However, higher concentrations of 2-DG also had some effects on normal cells (Fig. 5a, b, S7b, c), indicating that further consideration and optimization are needed for HK as a potential drug target. We next investigated whether targeting NPPS-HK1-glycolysis axis could sensitize KRAS-mutant cells to the targeted therapies. In H2030 cells bearing KRAS^G12C^ mutation, sotorasib (a KRAS^G12C^ inhibitor) synergized with Enpp-1-IN-1 or 2-DG to inhibit cell viability, as indicated by the CI values (Fig. 5e–g). These data indicate that pharmacologically targeting the NPPS-HK1-glycolysis axis profoundly impairs RAS-mutant cancers.

**Fig. 6:**
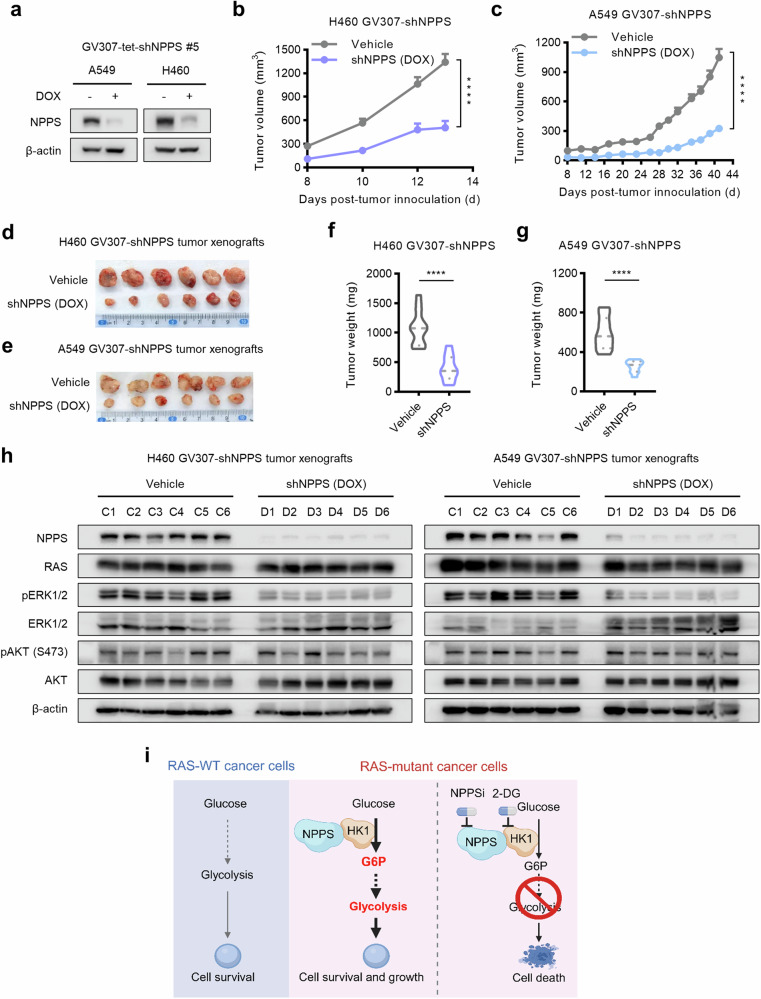
Targeting the NPPS delays tumorigenesis in vivo. **a** Knockdown efficacy of DOX-inducible shNPPS #5 A549 (left) and H460 (right) cell lines examined by Western blot analysis. The cells were treated with or without DOX (0.8 μg/mL) for 72 h. Growth curves (**b**, **c**), tumor volumes (**d**, **e**), and tumor weights (**f**, **g**) of H460 shNPPS-CDX and A549 shNPPS-CDX tumors. Water containing 2 mg/mL DOX (in 25 mg/mL sucrose) or vehicle control (25 mg/mL sucrose) was administered to each cohort. *n* = 12 for the vehicle group of H460 CDX tumors and *n* = 10 for the other groups. **h** Protein levels of NPPS and downstream signaling factors of RAS in H460 (left) and A549 (right) CDX tumors examined by Western blot analysis. Six tumor samples per group were randomly selected. **i** Schematic demonstration of the NPPS dependence in RAS mutant cancers. Created with https://www.BioRender.com. Data are shown as means ± SEMs (*n* = 3 replicates). ns, not significant; ****P  <  0.0001.

To determine in vivo whether NPPS could serve as a promising therapeutic target in RAS-mutant cancers, we used DOX-inducible shNPPS RAS-mutant cell lines to conditionally interfere with NPPS. After DOX treatment for 72 h, NPPS was markedly knocked down in vitro (Fig. 6a). H460 and A549 DOX-inducible shNPPS cells-derived xenograft (CDX) subcutaneous tumors in BALB/c *nu*/*nu* mice responded well to DOX-induced knockdown of NPPS compared to Vehicle, as evidenced by substantially reduced tumor growth (Fig. 6b, c), tumor volume (Fig. 6d, e), and tumor weight (Fig. 6f, g). In addition, DOX treatment did not cause body weight loss in the mice (Fig. S8a, b). Furthermore, continuous DOX treatment induced the knockdown of NPPS in tumors (Fig. 6h), recapitulating the in vitro results in which NPPS was knocked down in H460 and A549 shNPPS cells after DOX treatment (Fig. 6a). Compared with the vehicle control, the knockdown of NPPS resulted in the inhibition of phospho-ERK1/2 (pERK1/2), a canonical downstream signaling factor of RAS, indicating the inhibition of RAS-driven tumorigenic growth (Fig. 6h). Taken together, these data indicate that targeting NPPS exhibits therapeutic potential in RAS-mutant tumors in vivo.

## Discussion

With the advancement of molecular diagnostic technology, it has been discovered that gene mutations or abnormal expression play crucial roles in driving the development of cancers. The frequency of RAS mutations in cancer patients is approximately 30% [2, 4], but progress in targeted therapy for RAS mutations has been limited, with only KRAS^G12C^ inhibitors being developed and entering clinical use [5–7]. Therefore, the discovery of specific regulatory proteins or nodes for pan-RAS mutant tumors can provide potential strategies for RAS-driven cancers.

In our study, we observed a widespread upregulation of NPPS in RAS-mutant cancer cell lines and isogenic cell lines. Knockdown and pharmacological inhibition of NPPS selectively inhibited the survival of RAS-mutant cells while exerting minimal effects on normal cells. NPPS is a nucleotide pyrophosphatase that catalyzes the hydrolysis of triphosphate nucleotides into monophosphate nucleotides. Therefore, we investigated alterations in nucleotide metabolism in RAS-mutant cells and found no significant increase in these cells. Subsequent analyses via RNA-seq and untargeted metabolomics revealed significant alterations in glycolytic metabolism in RAS-mutant cells. Furthermore, both the seahorse assay and metabolomics analysis demonstrated substantial enhancement of glycolytic metabolism in RAS-mutant cells, and knockdown of NPPS effectively inhibited increased glycolysis in RAS-mutant cells.

Next, we aimed to explore how NPPS regulates glycolysis. Our previous studies revealed that some metabolic enzymes have nonmetabolic functions. For instance, aldo-keto reductase family 1 member B1 (AKR1B1) enhances glutathione synthesis by upregulating signal transducer and activator of transcription 3 (STAT3)-dependent transcription of solute carrier family 7 member 11 (SLC7A11) through its interaction with P-STAT3 [15]. Additionally, phosphoglycerate mutase 1 (PGAM1) interacts with α-smooth muscle actin (ACTA2) to regulate the invadopodia-like structures [17]. Based on our previous investigations, we conducted IP‒MS analysis to identify the proteins interacting with NPPS. The results suggested that the glycolysis-associated proteins HK1 and PFKP specifically interacted with NPPS in RAS-mutant cells. Co-IP assays confirmed the increased interaction between NPPS and HK1 in RAS-mutant cells, which was further substantiated in HEK293T and RAS-WT cells expressing Flag-NPPS. Furthermore, NPPS specifically interacted with the N-terminal region (aa 22–474) of HK1 to promote its enzymatic activity in RAS-mutant cells. SIRM analysis based on LC‒MS/MS revealed a significant increase in glycolytic intermediates in RAS-mutant cells, with G6P, the product of HK, exhibiting the greatest change. These findings indicated that NPPS interacted with HK1 to enhance its enzymatic activity, thereby promoting glycolysis. Thus, the inhibition of HK should have a similar effect as the suppression of NPPS. The results demonstrated that treatment with 2-DG, an HK1 inhibitor, selectively inhibited the cell viability and growth of RAS-mutant cells compared with normal cells and most RAS-WT cells, except for HCC827 cells. However, higher concentrations of 2-DG also had some effects on normal cells.

The NPPS inhibitor demonstrates a selective inhibition of RAS-mutant cells in vitro. However, owing to the higher IC_50_, we chose to construct inducible knockdown of NPPS cell lines for in vivo subcutaneous xenograft experiments, and the results showed that knockdown of NPPS significantly inhibited the tumorigenesis of RAS-mutant cells in vivo.

In summary, our study uncovers a novel mechanism that NPPS boosts glycolysis and promotes cell proliferation in RAS-mutant cancers through its interaction with HK1, a rate-limiting enzyme in glycolysis. Thus, targeting the NPPS-HK1-glycolysis axis can significantly suppress RAS-mutant tumors (Fig. 6i). This study provides a promising strategy for the targeted therapy of cancers harboring pan-RAS mutations.

## Supplementary information

Supplementary Figure S1

Supplementary Figure S2

Supplementary Figure S3

Supplementary Figure S4

Supplementary Figure S5

Supplementary Figure S6

Supplementary Figure S7

Supplementary Figure S8

Supplementary figure legend
