# Supplementary figures and images for: Dependence of NPPS creates a targetable vulnerability in RAS-mutant cancers
Source: Acta Pharmacol Sin. 2024 Nov 6;46(3):728–39. doi: 10.1038/s41401-024-01409-2 (PMC11845791; doi:10.1038/s41401-024-01409-2)

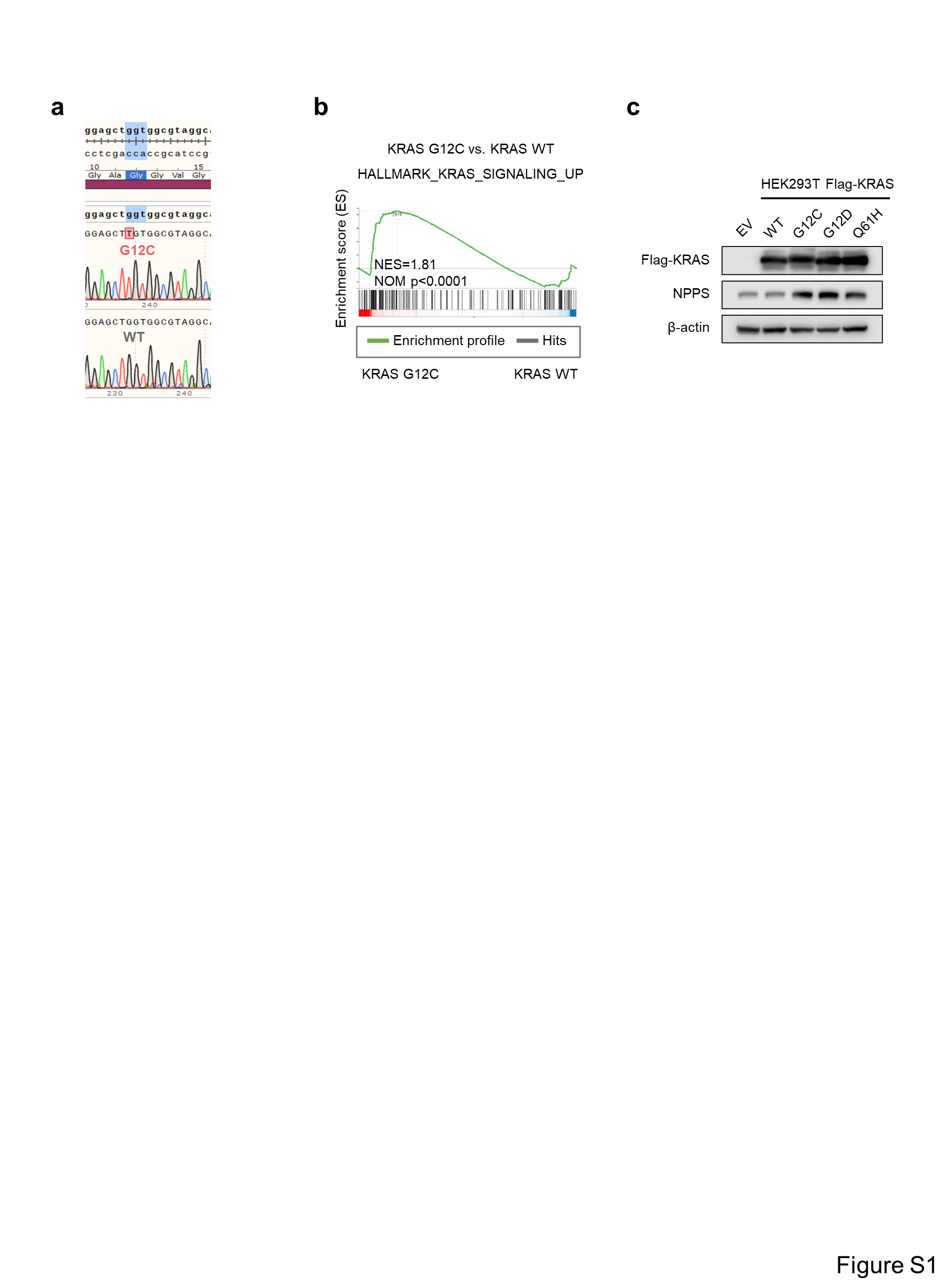

Supplement: Supplementary file 1 — Supplementary Figure S1 [file 41401_2024_1409_MOESM1_ESM.tif]

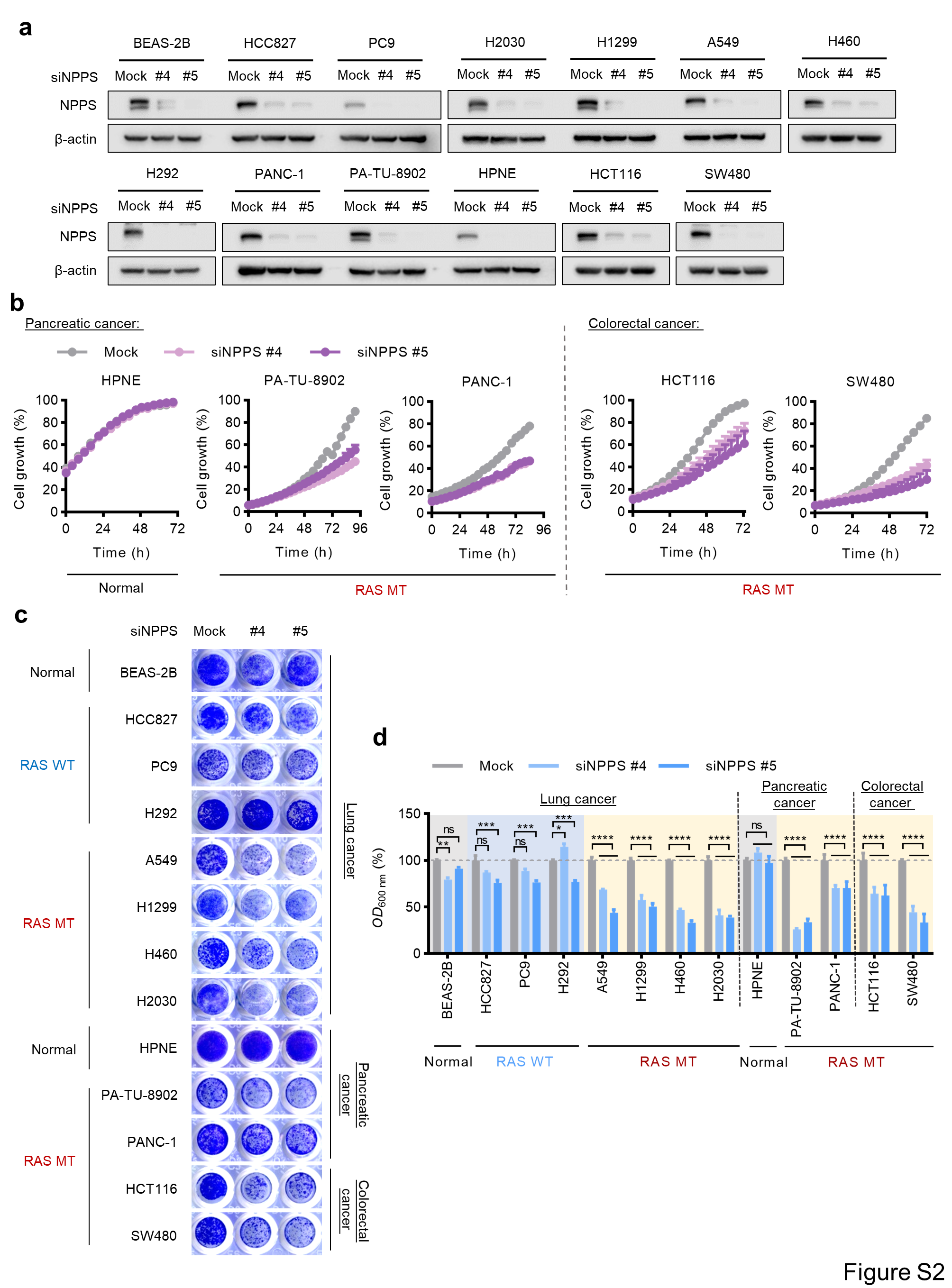

Supplement: Supplementary file 2 — Supplementary Figure S2 [file 41401_2024_1409_MOESM2_ESM.tif]

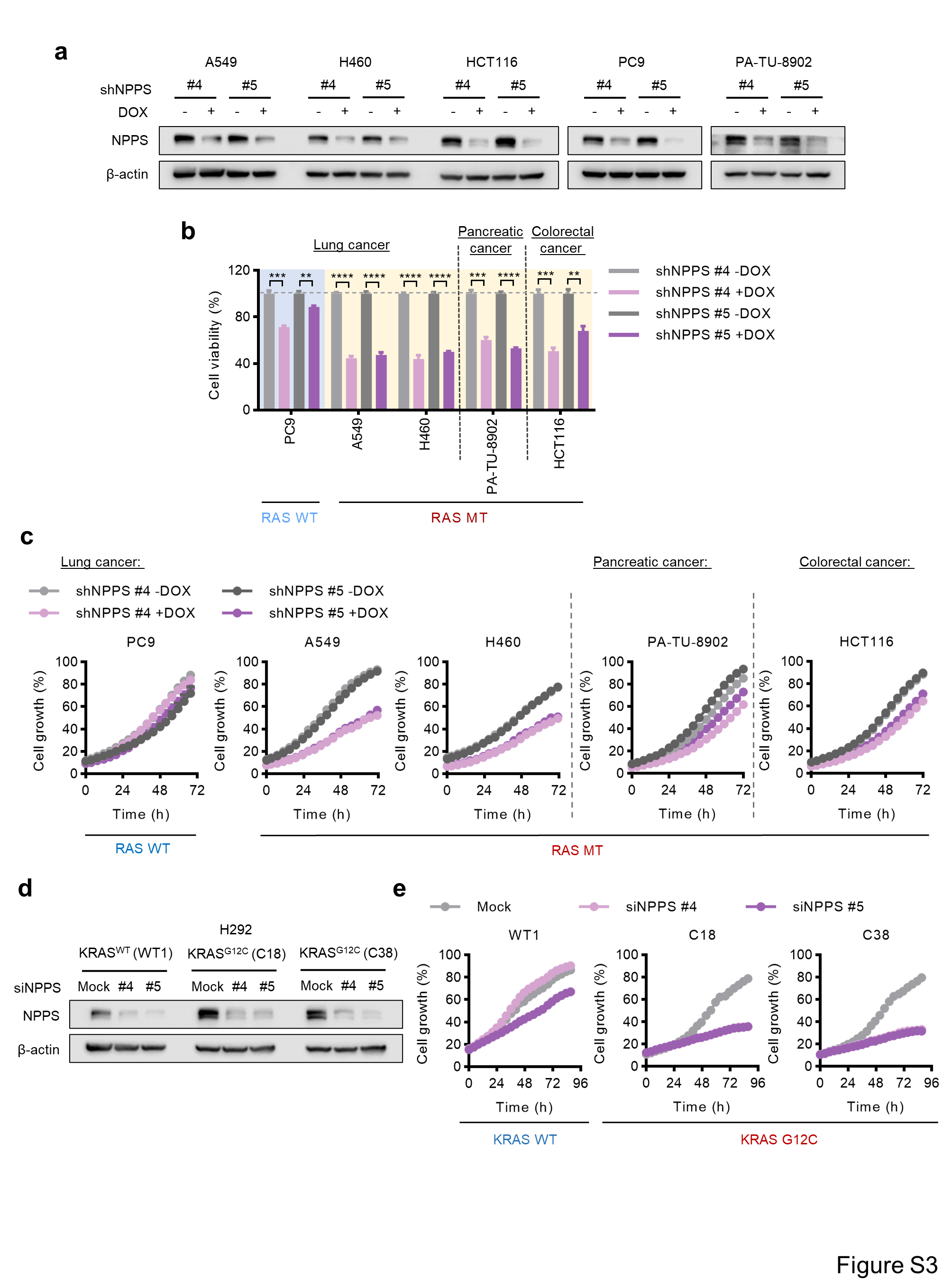

Supplement: Supplementary file 3 — Supplementary Figure S3 [file 41401_2024_1409_MOESM3_ESM.tif]

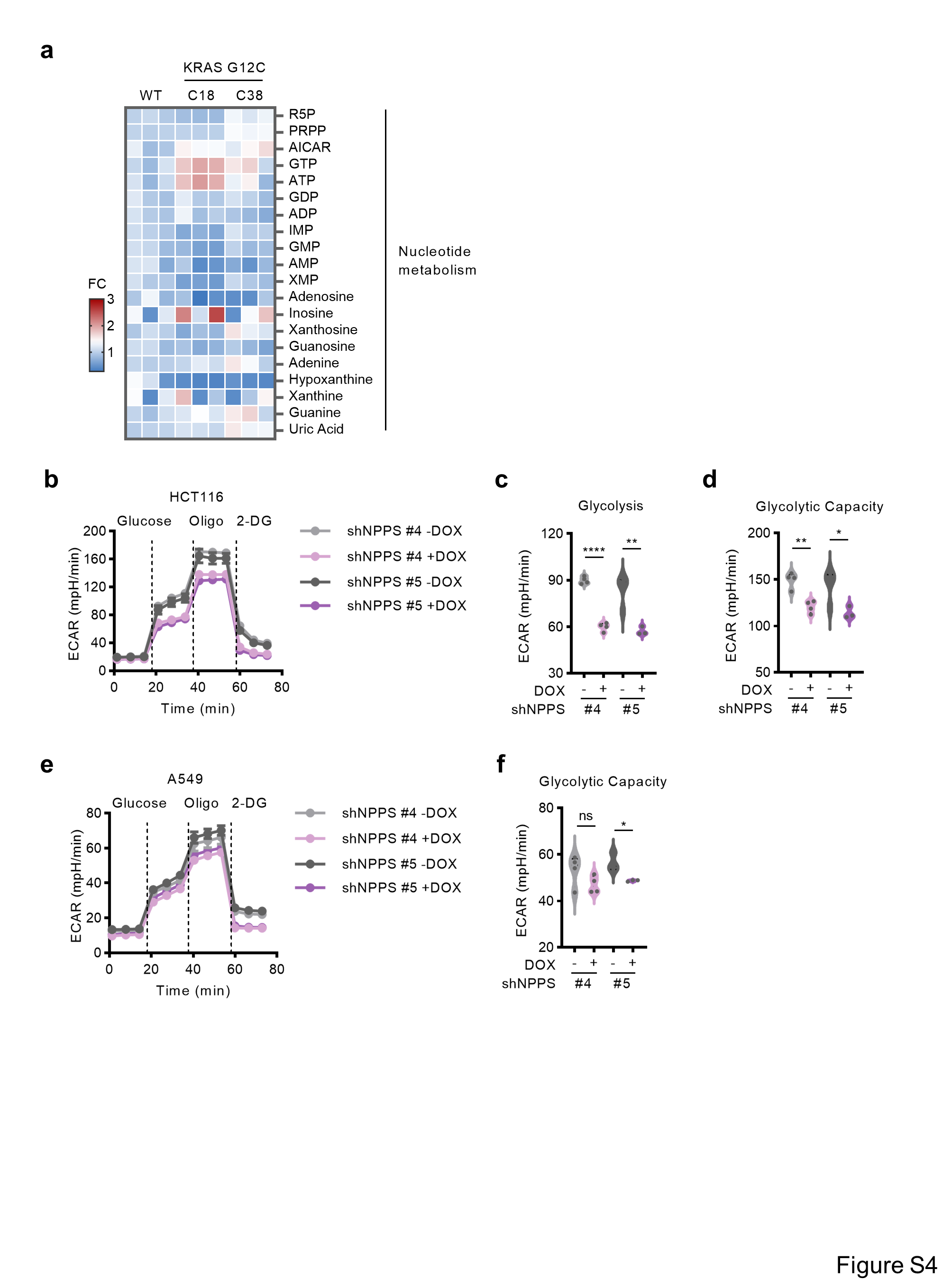

Supplement: Supplementary file 4 — Supplementary Figure S4 [file 41401_2024_1409_MOESM4_ESM.tif]

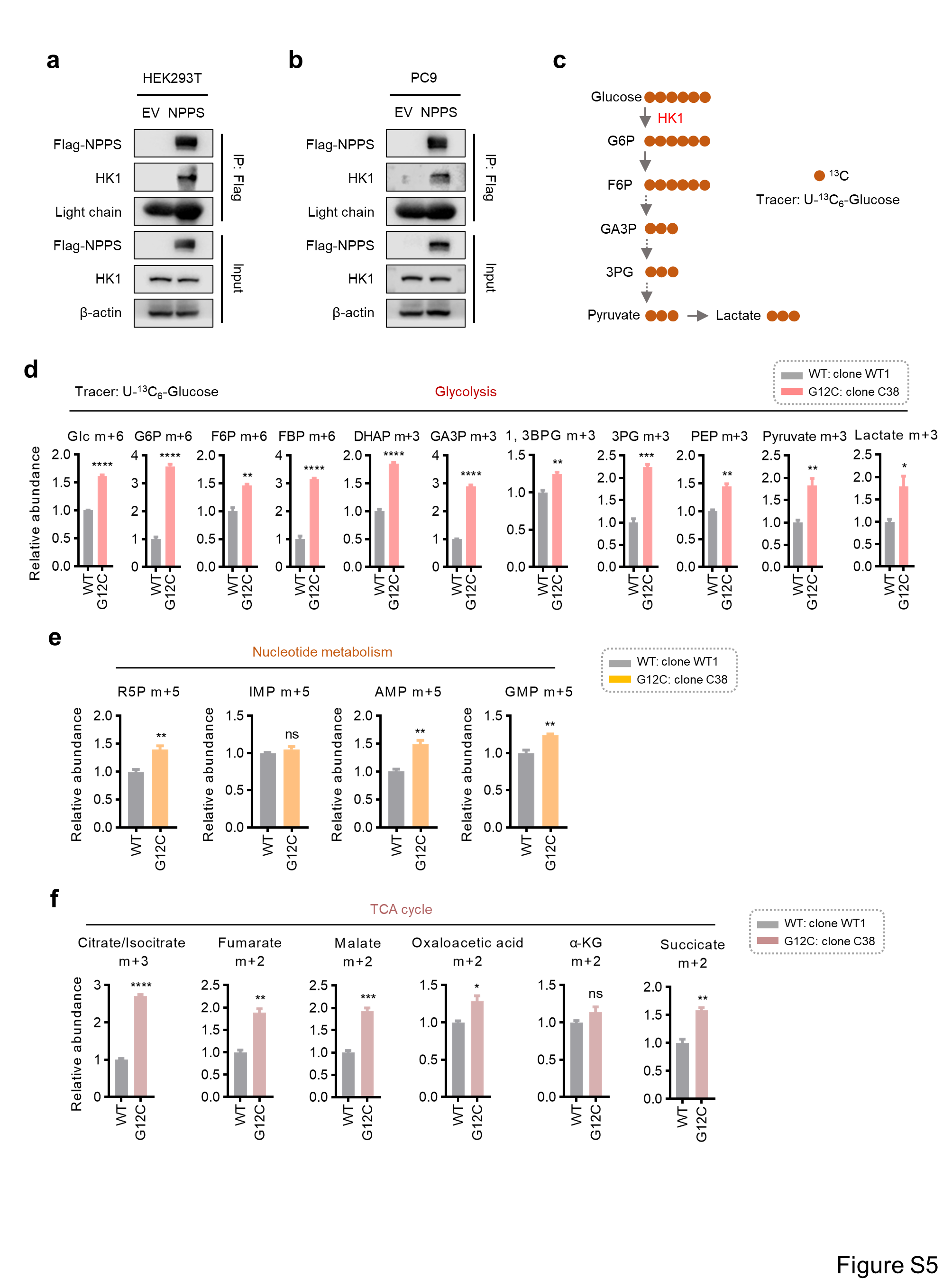

Supplement: Supplementary file 5 — Supplementary Figure S5 [file 41401_2024_1409_MOESM5_ESM.tif]

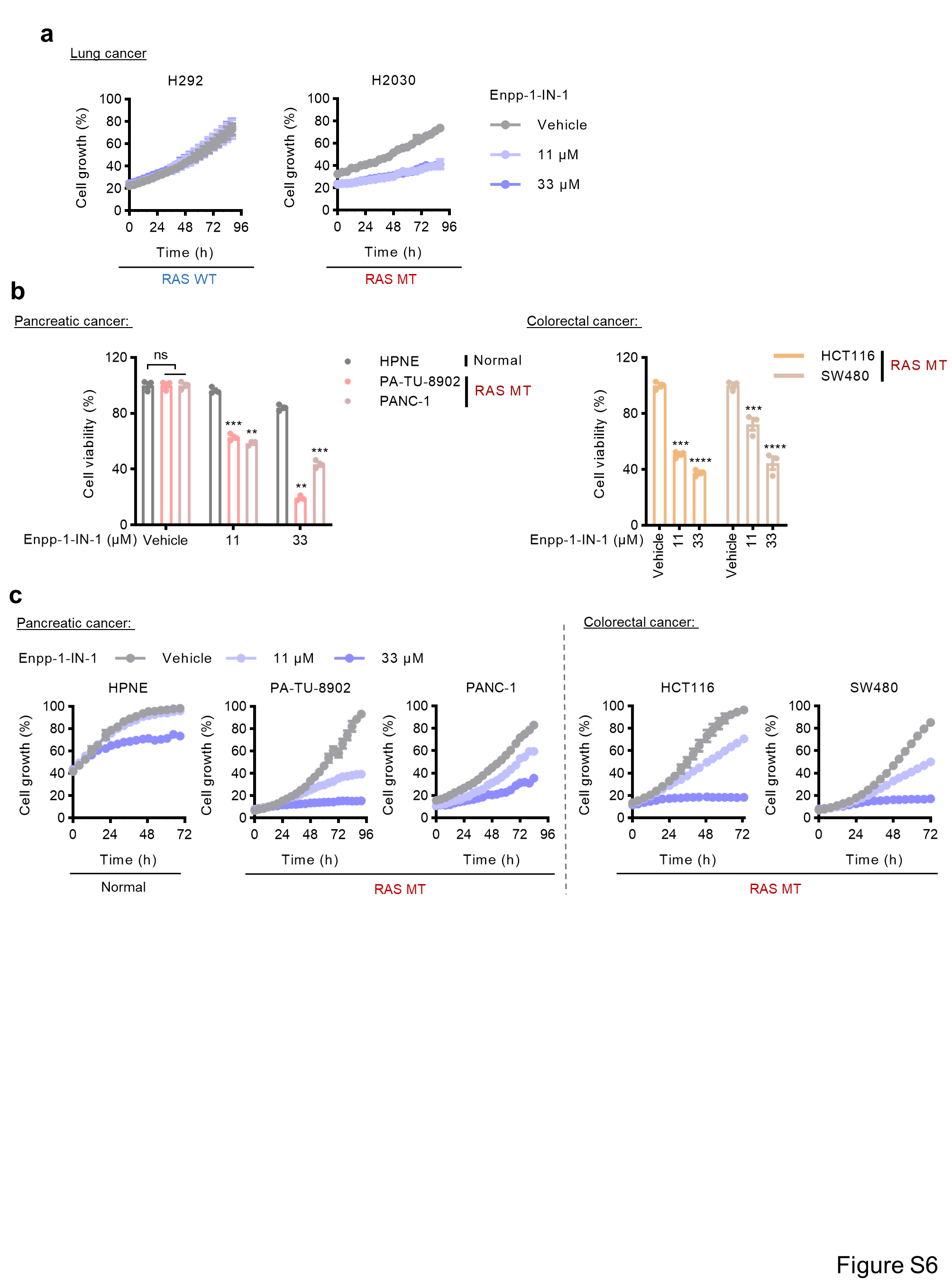

Supplement: Supplementary file 6 — Supplementary Figure S6 [file 41401_2024_1409_MOESM6_ESM.tif]

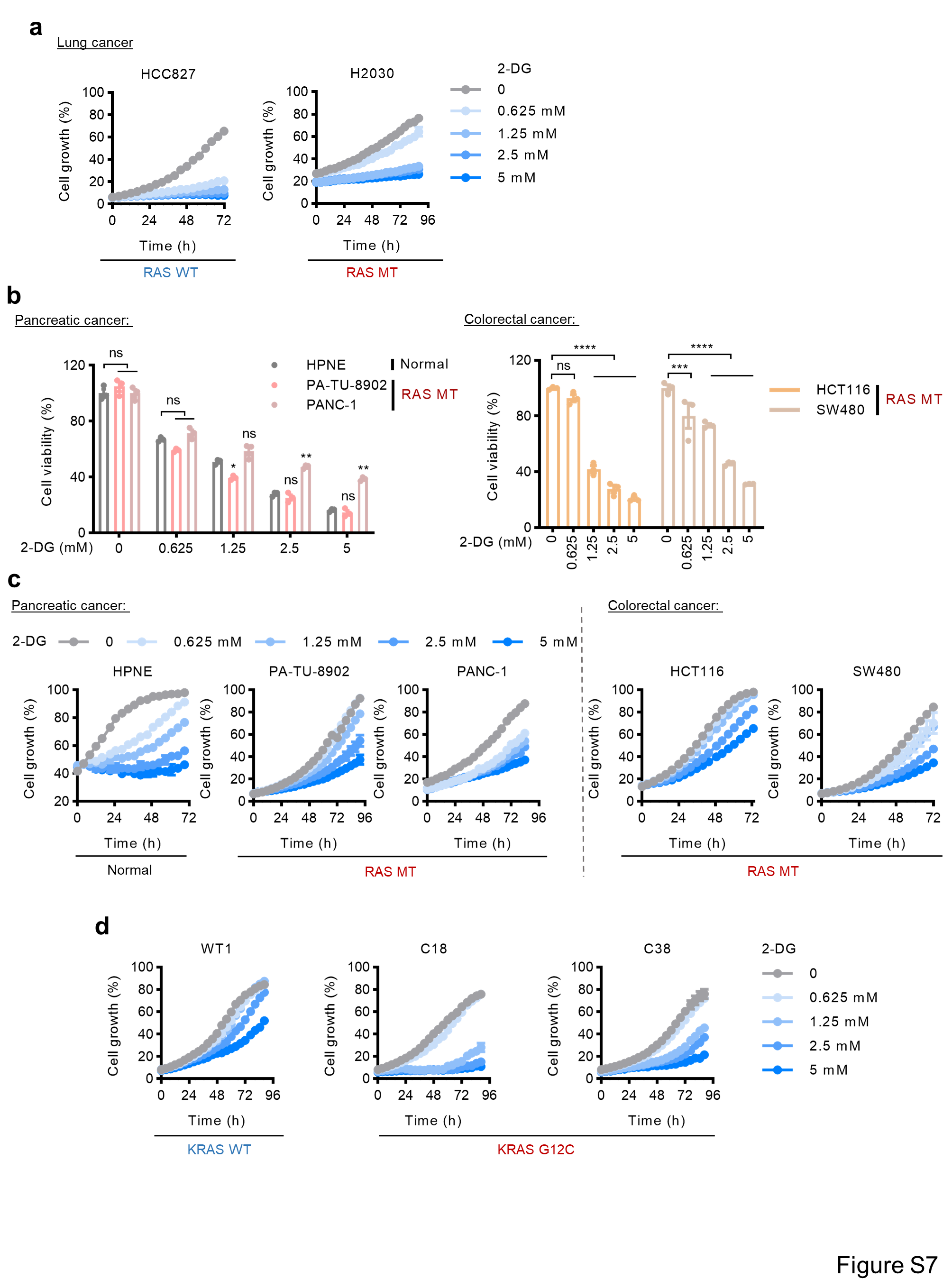

Supplement: Supplementary file 7 — Supplementary Figure S7 [file 41401_2024_1409_MOESM7_ESM.tif]

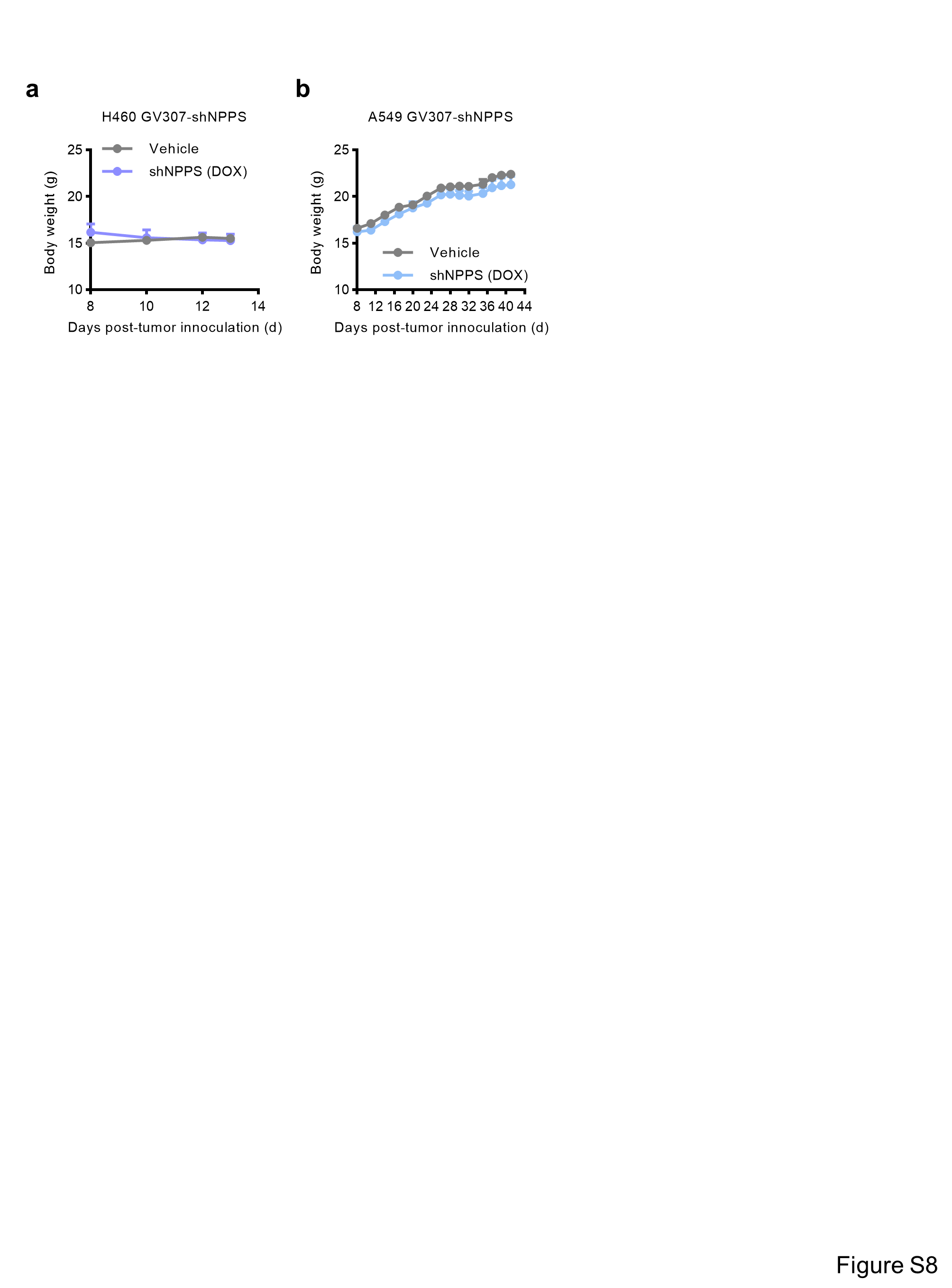

Supplement: Supplementary file 8 — Supplementary Figure S8 [file 41401_2024_1409_MOESM8_ESM.tif]
