# Supplementary material for: Dependence of NPPS creates a targetable vulnerability in RAS-mutant cancers
Source: Acta Pharmacol Sin. 2024 Nov 6;46(3):728–39. doi: 10.1038/s41401-024-01409-2 (PMC11845791; doi:10.1038/s41401-024-01409-2)
Supplement: Supplementary file 9 — Supplementary figure legend [file 41401_2024_1409_MOESM9_ESM.docx]

# Dependence of NPPS creates a targetable vulnerability in RAS-mutant cancers

# Supplementary figure legend

## Fig. S1: NPPS is upregulated in RAS-mutant cells

**a** Sanger sequencing of H292 isogenic cells. Data were analyzed by the SnapGene software. **b** GSEA of RNA-seq datasets indicated a KRAS signaling up hallmark in H292^KRAS G12C^ cells compared with H292^KRAS WT^ cells. **c** Protein levels of NPPS in HEK293T cells expressing empty vector (EV), wild-type KRAS and indicated KRAS variants examined by Western blot analysis.

## Fig. S2: RAS-mutant cell lines are more dependent on NPPS

**a** Knockdown efficacy of NPPS siRNAs examined by Western blot analysis. The cells were transfected with NPPS siRNAs (20 nM) or mock control for 72 hours. **b** Growth curves of cell lines after knockdown of NPPS. The cells were transfected with NPPS siRNAs (20 nM) or mock control for 72–96 hours and monitored by IncuCyte ZOOM system every 4 hours. **c, d** Cell growth analysis of cell lines after knockdown of NPPS. The cells were transfected with NPPS siRNAs (20 nM) or mock control for 72 hours and then cell growth was measured by the crystal violet assay. Data are shown as means ± SEMs (n=3 replicates). ns, not significant; *P < 0.05, **P < 0.01, ***P < 0.001, ****P < 0.0001.

## Fig. S3: Knockdown of NPPS selectively suppresses RAS-mutant cells

**a** The knockdown efficacy of DOX-inducible shNPPS #4 and #5 in indicated cells examined by Western blot analysis. The cells were treated with or without DOX (0.8 μg/mL) for 72 hours. **b** Cell viability of a panel of cell lines after knockdown of NPPS. The cells were treated with or without DOX (0.8 μg/mL) for 72 hours and then cell viability was detected by CCK-8. **c** Growth curves of cell lines after knockdown of NPPS. The cells were treated with or without DOX (0.8 μg/mL) for 72 hours and monitored by IncuCyte ZOOM system every 4 hours. **d** Knockdown efficacy of NPPS siRNAs examined by Western blot analysis. H292^KRAS WT^ cells and H292^KRAS G12C^ cells were transfected with NPPS siRNAs (20 nM) or mock control for 72 hours. **e** Growth curves of H292^KRAS WT^ cells and H292^KRAS G12C^ cells after knockdown of NPPS. The cells were transfected with NPPS siRNAs (20 nM) or mock control for 96 hours and monitored by IncuCyte ZOOM system every 4 hours. Data are shown as means ± SEMs (n=3 replicates). ns, not significant; *P < 0.05, **P < 0.01, ***P < 0.001, ****P < 0.0001.

## Fig. S4: Hyperglycolysis in RAS-mutant cells depends on NPPS

**a** Heatmap of the metabolites involved in nucleotide metabolism in H292 isogenic cells determined by LC-MS/MS analysis. **b-d** Glycolysis of HCT116 cells after knockdown of NPPS. The cells were treated with or without DOX (0.8 μg/mL) for 72 hours and then the ECAR was measured using Seahorse XFe96 Analyzer. **e, f** Glycolysis of A549 cells after knockdown of NPPS. Cells were treated with or without DOX (0.8 μg/mL) for 72 hours and then the ECAR was measured using Seahorse XFe96 Analyzer. Data are shown as means ± SEMs (n=3 replicates). ns, not significant; *P < 0.05, **P < 0.01, ***P < 0.001, ****P < 0.0001.

## Fig. S5: Function of NPPS in RAS-mutant cells is independent of its canonical nucleotide-metabolizing activity

**a, b** Anti-Flag Co-IP assays of the NPPS-HK1 interaction in HEK293T (**a**) and PC9 cells (**b**) expressing Flag-NPPS compared with those expressing the empty vector (EV). **c** Schematic diagram of U-^13^C_6_-glucose-derived, labeled intermediates in glycolytic pathway determined by LC‒MS/MS-based SIRM analysis. **d** Relative levels of glycolytic intermediates in H292^KRAS G12C^ cells (clone C38) compared with H292^KRAS WT^ cells (clone WT1) determined by the SIRM analysis. The cells were cultured in glucose-free RPMI 1640 medium supplemented with 10% FBS and 11 mM U-^13^C_6_-glucose for 6 h. **e, f** Relative levels of the U-^13^C_6_-glucose-derived, labeled metabolites involved in nucleotide synthesis (**e**) and the TCA cycle (**f**) in H292^KRAS G12C^ cells (clone C38) compared with H292^KRAS WT^ cells (clone WT1) determined by LC‒MS /MS-based SIRM analysis. Data are shown as means ± SEMs (n=3 replicates). ns, not significant; *P < 0.05, **P < 0.01, ***P < 0.001, ****P < 0.0001.

## Fig. S6: Inhibition of NPPS suppresses RAS-mutant cells

**a** Growth curves of H292 and H2030 cells after treatment with Enpp-1-IN-1 at the indicated concentrations. Cells were treated with Enpp-1-IN-1 for 96 hours and monitored by IncuCyte ZOOM system every 4 hours. **b** Cell viability of a panel of cell lines after treatment with Enpp-1-IN-1 at the indicated concentrations. The cells were treated with Enpp-1-IN-1 for 72 hours and then cell viability was measured using CCK-8. **c** Growth curves of cell lines after treatment with Enpp-1-IN-1 at the indicated concentrations. The cells were treated with Enpp-1-IN-1 for 72–96 hours and monitored by IncuCyte ZOOM system every 4 hours. Data are shown as means ± SEMs (n=3 replicates). ns, not significant; *P < 0.05, **P < 0.01, ***P < 0.001, ****P < 0.0001.

## Fig. S7: Targeting HK1 suppresses RAS-mutant cells

**a** Growth curves of HCC827 and H2030 cells after treatment with 2-DG at the indicated concentrations. The cells were treated with 2-DG for 72‒96 hours and monitored by IncuCyte ZOOM system every 4 hours. **b** Cell viability of a panel of cell lines after treatment with 2-DG at the indicated concentrations. The cells were treated with 2-DG for 72 hours and then cell viability was measured using CCK-8. **c** Growth curves of cell lines after treatment with 2-DG at the indicated concentrations. The cells were treated with 2-DG for 72–96 hours and monitored by IncuCyte ZOOM system every 4 hours. **d** Growth curves of H292^KRAS WT^ cells and H292^KRAS G12C^ cells after treatment with 2-DG at the indicated concentrations. Cells were treated with 2-DG for 96 hours and monitored by IncuCyte ZOOM system every 4 hours. Data are shown as means ± SEMs (n=3 replicates). ns, not significant; *P < 0.05, **P < 0.01, ***P < 0.001, ****P < 0.0001.

## Fig. S8: Targeting NPPS inhibits tumorigenesis *in vivo*

**a, b** Body weights of BALB/c nu/nu mice with H460 (**a**) and A549 (**b**) CDX tumors. Water containing 2 mg/mL DOX (in 25 mg/mL sucrose) or vehicle control (25 mg/mL sucrose) was administrated to each cohort. n=6 for the vehicle group of H460 CDX and n=5 for the other groups.
